# Psychosocial hazards, posttraumatic stress disorder, complex posttraumatic stress disorder, depression, and anxiety in the U.K. rail industry: A cross‐sectional study

**DOI:** 10.1002/jts.22846

**Published:** 2022-06-22

**Authors:** Laurence A. Carnall, Oliver Mason, Michelle O'Sullivan, Robert Patton

**Affiliations:** ^1^ School of Psychology University of Surrey Guildford United Kingdom; ^2^ Rail Safety and Standards Board London United Kingdom

## Abstract

This study examined posttraumatic stress disorder (PTSD), complex PTSD, depression, and anxiety among U.K. rail workers. A cross‐sectional survey examining exposure to seven psychosocial hazards (bullying/harassment; verbal abuse; physical and sexual assault; and hearing about, seeing the aftermath of, or witnessing a fatality), working conditions, physical health, and the impact of COVID‐19 was administered to 3,912 participants. Outcome measures were the ITQ, PHQ‐9, and GAD‐7. Among trauma‐exposed participants, 24.3% met the criteria for PTSD or CPTSD; 38.6% and 29.2% of all participants scored in the moderate‐to‐severe range on the PHQ‐9 and GAD‐7, respectively. Data were analyzed using logistic and linear regression. Bullying/harassment was positively associated with GAD‐7 scores, *f* ^2^ = .001, and PTSD and CPTSD, *OR*s = 1.83–2.02. Hearing about and witnessing a fatality were associated with PTSD and CPTSD, *OR*s = 1.77–2.10. Poorer ergonomics at work were positively associated with PHQ‐9 and GAD‐7 scores, *f* ^2^ = .001. Higher job satisfaction was associated with lower odds of PTSD and CPTSD, *OR*s = 0.87–0.91, and negatively associated with PHQ‐9 and GAD‐7 scores, *f* ^2^ = .008–.01. Work intensity was associated with PTSD and CPTSD, *OR*s = 1.79–1.83, and positively associated with PHQ‐9 and GAD‐7 scores, *f* ^2^ = .02–.03. Reporting more physical health problems was associated with PTSD, *OR* = 1.07, and positively associated with GAD‐7 and PHQ‐9 scores, *f* ^2^ = .008–.01. The results suggest bullying/harassment and work intensity are important variables in employee mental health and could drive future research and industry initiatives.

The diagnostic category of posttraumatic stress disorder (PTSD) is historically intertwined with the study of occupational exposure to stressful situations. Although it originally emerged in a military context (Crocq & Crocq, 2000), the remit has since widened. A review by Skogstad and colleagues (2013) highlighted that police officers, firefighters, and ambulance crews are frequently exposed to traumatic events, with high rates of PTSD found among these workers. Studies have suggested PTSD prevalence rates of 11% in ambulance workers (Petrie et al., 2018), 20.6% in front‐line police officers (Brewin et al., 2020), and 23.9% in firefighters (Langtry et al., 2021). Skogstad and colleagues (2013) argued that occupations in which trauma exposure is commonplace may provide more training to increase resilience; for instance, they found that professional firefighters appeared to cope better with traumatic events than volunteers. This finding may be of particular interest in light of other, perhaps less obvious, professions that may confer an elevated risk of exposure to potentially traumatic events, such as train drivers.

Train drivers, along with other railway industry workers, can be exposed to a range of stressful and threatening situations. Their roles also fulfill a crucial public service and often require a high level of skill and attention to detail, making mental health difficulties a potential concern with regard to both safety (Jeon et al., 2014) and employee well‐being. Most previous research has focused on train crews exposed to deaths and near‐misses due to “person under train” (PUT) incidents, as these can lead to severe outcomes, such as PTSD (Weiss & Farrell, 2006). One study found that drivers experienced an average of 1.8 PUTs in their career, with 44% of the sample classified as suffering from moderate‐to‐severe PTSD (Mehnert et al., 2012). Another study found that 17.1% of PUT‐exposed drivers presented with symptoms of PTSD and depression 1‐month postincident (Tranah & Farmer, 1994). A review of suicide on the tube network (i.e., underground public transportation and train system) in the United Kingdom deemed drivers’ PTSD symptoms comparable to those suffered by Vietnam War veterans (Cocks, 1989).

Although research into the impact of exposure to suicides and PUTs on train drivers is important, there is a wider range of trauma exposure relevant to mental health in this population. The U.K. Health and Safety Executive (HSE) termed these “psychosocial hazards,” reflecting any aspect of work that could detrimentally affect an employee's well‐being (HSE, 2001). Along with general psychosocial hazards associated with a range of professions (see Eurofund, 2015), rail industry workers can be exposed to verbal abuse, threatening behavior, violence or assault, witnessing death, and hearing the details of death relayed by another person (Rail Safety and Standards Board, 2019).

Another factor of interest is the quality of the workplace environment. Previous research in emergency services has demonstrated that job satisfaction and support from colleagues can be protective against PTSD (Somville et al., 2016). Similar findings have been observed among firefighters (e.g., Meyer et al., 2012) as well as health care workers exposed to viral outbreaks and pandemics (Carmassi et al., 2020). In their review on risk and resilience factors in PTSD, Lauth‐Lebens and Lauth (2016) argued that social support appeared to be the most important posttraumatic risk factor, whereas other researchers have noted the importance of workplace social support (Brooks et al., 2019) and other factors, such as administrative procedures, workload, and resources (McFarlane & Bryant, 2007).

To our knowledge, no study to date has examined the mental health consequences of exposure to a wide range of potentially traumatic events and hazards, nor the impact of social support and working conditions, among front‐line rail industry workers. One previous study examined PTSD and complex PTSD (CPTSD) in serving officers employed by the U.K. Police Service. Brewin and colleagues (2020) examined a broad range of potential exposures and covariates, arguing that there was a lack of large‐scale research in this area. The authors found that 85% of officers reported exposure to traumatic events, with 20.6% meeting the criteria for PTSD or CPTSD (Brewin et al., 2020). These findings highlight that high‐profile professions may, in some cases, be understudied, and that rates of PTSD, CPTSD, and, presumably, other mental health conditions, are likely to be elevated in occupations with a higher risk of trauma exposure. This underlines the impact large cross‐sectional studies can have in bringing these issues to light and directing further research.

Given the risk of additional trauma exposure alongside PUTs, the importance of the workplace environment, and the lack of previous research in this area, the current study implemented a similar design to Brewin and colleagues (2020) to investigate trauma exposure and mental health in the U.K. rail industry. We aimed to investigate the rate of exposure to seven psychosocial hazards relevant to rail‐industry workers based on industry data and anecdotal reports: bullying and harassment, verbal abuse, physical assault, sexual assault, hearing about a fatality on the railway, seeing the aftermath of a fatality, and witnessing a fatality. We also assessed aspects of workplace conditions, individual factors, demographic characteristics, and the impact of the COVID‐19 pandemic. Finally, we investigated the rates of symptoms of PTSD and CPTSD, depression, and generalized anxiety disorder in the sample.

We had three main hypotheses. First, we expected that psychosocial hazards would be associated with more severe symptoms of depression and anxiety and a higher likelihood of meeting the diagnostic thresholds for PTSD and CPTSD. Second, based on evidence that cumulative exposure to traumatic events is associated with increased symptoms of depression and PTSD (Martin et al., 2013), we hypothesized that the total number of exposures to each hazard would be independently associated with these outcomes. Finally, we posited that poorer working conditions would contribute to the associations between exposure and mental health symptoms, based on findings outlined previously. Covariates measuring various impacts of the COVID‐19 pandemic were included to control for the pandemic's clear impact on mental health (e.g., Jia et al., 2020; Zhang et al., 2021); we included other covariates related to physical health for similar reasons (e.g., Meader et al., 2011).

## METHOD

### Participants

All front‐line railway industry workers employed by participating organizations in the United Kingdom and who were at least 18 years of age were eligible for study participation; there were no other inclusion criteria. In total, 3,912 participants completed the survey. The estimated workforce of participating companies is 120,000, giving an estimated response rate of 3.3%. The mean participant age was 44.62 years (*SD* = 11.24, range: 18–74 years). In total, 72.3% of participants were male, 25.6% were female 0.2% were intersex, and 1.9% declined to answer. The mean age of male participants was 45.57 years (*SD* = 11.11), and the mean age of female participants was 42.23 years (*SD* = 11.22). Regarding ethnicity, 89.9% of the sample identified as White/White British, 2.4% as Asian or Asian British (i.e., Indian, Pakistani, Bangladeshi, Chinese, or “other”), 2.2% as Black or Black British (i.e., Caribbean, African or “other”), 1.6% as being of mixed or multiple ethnicities, 0.8% as “other”, and 3.1% declined to answer. For sexuality, 85.9% identified as heterosexual, 6.4% as gay or lesbian, 3.1% as bisexual, 0.7% as “other,” and 3.8% declined to answer. The vast majority of participants reported no disabilities (85.4%), 11.0% said they considered themselves to have a disability, and 3.6% declined to answer. In total, 18.0% of the sample was single, 25.3% reported being in a relationship or cohabiting, 48.3% were married, 5.5% were divorced or separated, 0.5% were widowed, and 2.5% declined to answer.

### Procedure

This study was conducted as a single point in time survey administered online via Qualtrics (Provo, UT, United States). The study was given ethical approval by the University of Surrey Faculty of Health and Medical Sciences Ethics Committee. All employees who met the inclusion criteria were sent an email invitation that included basic information on the study aims and contained an anonymous link to the online survey. All initial emails were sent by companies to their respective eligible employees, and three subsequent reminder emails were sent to all potential participants. There were no material incentives for participation. After clicking the email link, participants were taken to the information page and consent form. Once they provided informed consent, participants completed all measures except the International Trauma Questionnaire (ITQ; Cloitre et al., 2018), which, for ethical and validity reasons, was only completed if a participant confirmed they had experienced a potentially traumatic event, based on a brief definition of such events. After finishing the survey, participants were shown a page containing information on sources of support should they feel they needed it. Data were collected only once a participant clicked to confirm they wished to submit their data, and participants could opt‐out at any stage before completion. As the data were fully anonymized, including Internet Protocol (IP) addresses, participants could not opt‐out once they had submitted. This was made clear on the information page and in the consent form.

### Measures

#### Demographic variables, occupational characteristics, and psychosocial hazards

Information on demographic characteristics, including age, sex, ethnicity, and marital status, as well as professional characteristics, including occupation or role, length of time employed in the rail industry (in months and years), and employee job security (secure, temporary, or 0 hr contract). The survey also included items related to exposure to psychosocial hazards, including the estimated date of most recent exposure and estimated frequency.

#### Physical health

Physical health was assessed using a five‐response rating of physical health ranging from *very poor* to *very good*. Participants were also given a list of physical health problems and asked to indicate (i.e., endorse) whether they were or had experienced each, and a final item asked participants to estimate their total number of past‐year sickness absences.

#### Trauma exposure

The ITQ (Cloitre et al., 2018) is a brief measure of PTSD and CPTSD, with a diagnostic algorithm based on the criteria in the 11th revision of the *International Statistical Classification of Diseases and Related Health Problems* (*ICD‐11*; World Health Organization [WHO], 2019) that includes three possible outcomes: no diagnosis, PTSD, or CPTSD. The ITQ includes 18 items that are rated on a 5‐point Likert‐type scale of 0 (*not at all*) to 4 (*extremely*). Nine items examine intrusive thoughts and memories, avoidance, hypervigilance, and the impact of these symptoms on the person's relationships, social and work life. The other nine items cover emotional difficulties and beliefs about the self as well as factors associated with CPTSD and their impact on relationships, social life, and work life. The ITQ was validated in a large study using trauma‐exposed clinical and community samples from the United Kingdom and demonstrated diagnostic rates in line with previous estimates (Cloitre et al., 2018). The measure demonstrated good internal consistency in a large population‐based study (Cronbach's α = .89, Cloitre et al., 2019). In the present sample, internal consistency was excellent, Cronbach's α = .95.

#### Depressive symptoms

The nine‐item Patient Health Questionnaire (PHQ‐9; Kroenke et al., 2001) is a brief measure of depressive symptoms that is based on criteria from the fourth edition of the *Diagnostic and Statistical Manual of Mental Disorders* (*DSM‐IV*; American Psychiatric Association [APA], 2000). Items are scored on a Likert‐type scale ranging from 0 (*not at all*) to 3 (*nearly every day*), with total possible scores ranging from 0 to 27. The measure has demonstrated good internal consistency (Cronbach's α = .89) and good test–retest reliability (*r* = .84 at a 48‐hr interval). Criterion validity assessed by structured psychiatric interview compared to various cutoff scores on the PHQ‐9 has shown that a cutoff score of 9 or higher on the PHQ‐9 has a sensitivity of 95% and specificity of 84% to detect a major depressive episode; a cutoff of 15 or higher demonstrated a sensitivity of 68% and specificity of 95% (Kroenke et al., 2001). In the present study's sample, internal consistency was very good, Cronbach's α = .92.

#### Anxiety symptoms

The seven‐item Generalized Anxiety Disorder scale (GAD‐7; Spitzer et al., 2006) is a brief screening measure for GAD based on *DSM‐IV* criteria. Items are rated on a Likert‐type scale ranging from 0 (*not at all*) to 3 (*nearly every day*), with total possible scores ranging from 0 to 21. The GAD‐7 was originally developed and validated for primary care settings in a large U.S. and demonstrated good internal reliability (Cronbach's α = .92) and test–retest reliability (*r* = .83; Spitzer et al., 2006). Criterion validity was measured against mental health diagnosis; at a cutoff score of 8 or higher, the GAD‐7 was found to have a sensitivity of 92% and specificity of 76%, whereas at 15 points or higher, sensitivity and specificity were 48% and 95%, respectively (Spitzer et al., 2006). In the present sample, internal consistency was very good, Cronbach's α = .94.

#### Working conditions

The present study used a selection of Likert‐scale–rated and forced‐choice items from the European Working Conditions Survey (EWCS; Eurofund, 2015) related to working conditions, support from colleagues and institutions, satisfaction with work, and general physical health. The Likert‐scale items represent seven subscales, which were used in the regression analysis: Physical Environment, Physical Ergonomics, and Work Intensity, for which higher scores indicate worse conditions, as well as Social Environment, Skills and Autonomy, Work Time Quality, and Working Life Perspectives, for which higher scores indicate better conditions (Eurofund, 2015). For the present study, subscale scores were calculated by taking the sum of all relevant item scores in each category. To our knowledge, there are no studies available on the psychometric properties of the specific items used here; however, the subscales have overall construct validity with indices of health and well‐being (Eurofund, 2017). In the present sample, the internal consistency of the Likert‐scale items was acceptable, Cronbach's α = .75.

#### COVID‐19–related questions

A block of questions related to the COVID‐19 pandemic was included in the survey. These items were based on previous research in a sample of nursing staff working during the pandemic in China (Kang et al., 2020) and asked whether participants had been diagnosed with COVID‐19, with three options to indicate they had either tested positive, had symptoms but not been tested, or that they did not think they ever had COVID‐19. Three further questions asked whether household members, friends, or colleagues had contracted suspected COVID‐19 (i.e., “Has anyone in your household had suspected COVID‐19?”), with four forced‐choice responses indicating no suspected infection, infection and the need to isolate, infection and hospitalization, and death following infection. Unfortunately, there are no data available on the psychometric properties of these items. Cronbach's alpha was not calculated as these items did not share unidimensionality; it was assumed that infection status between participants and their colleagues and friends would not be consistent given the social distancing policies in place during data collection. Participants were also asked to indicate whether they felt their mental health had been affected by the pandemic.

### Data analysis

All analyses were conducted in SPSS (Version 27). Descriptive statistics for demographic data were compiled to demonstrate sample characteristics and rates for the seven main psychosocial hazards and mental health outcomes. Variables for the regression were entered blockwise, with the main predictors entered alone to allow us to first investigate raw associations. Demographic characteristics were entered second, followed by variables for working conditions, physical health, COVID‐19, and, finally, the total number of exposures to each psychosocial hazard. Blockwise entry was used to demonstrate how variable groups that shared theoretically related constructs contributed to the overall model, with demographic characteristics representing the first additional block to control for these variable groups in all later tests of model fit. Subsequent entry order was based on the assumption that each block could provide a significant contribution, at the point it was entered, over previously included variables. The order of entry did not impact the final model odds ratios (*OR*s) and coefficients because variable deletion and inclusion techniques were not used. Linear regression was chosen for PHQ‐9 and GAD‐7 scores, and logistic regression was chosen for PTSD and CPTSD. For the logistic regressions, interval variables were computed into *z* scores to allow for the standardization of odds ratios, with the exception of the total number of physical health problems.

Missing data were handled via listwise exclusion. No form of imputation was used. Almost all items contained some missing data. The amount of missing data per item included in the regression models ranged from 0.1% to 6.0% (*Mdn* = 0.56, *M* = 0.86, *SD* = 1.00). The percentage of participants excluded from regressions listwise was 18.3%. Little's Missing Completely at Random test did not support the hypothesis that data were missing completely at random, *p* < .001. Participants with and without missing data were compared using independent samples *t* tests for age and Pearson chi‐square tests for other demographic variables. Participants with missing values did not differ by age, *t*(3,886) = −0.37, *p* = .713. However, chi‐square tests were significant for sex, χ^2^(3, *N* = 3,905) = 51.37, *p* < .001; sexual orientation, χ^2^(4, *N* = 3,904) = 63.06, *p* < .001; marital status, χ^2^(5, *N* = 3,906) = 67.91, *p* < .001; and disability status, χ^2^(2, *N* = 3,897) = 49.58, *p* < .001, with participants selecting “prefer not to say” showing the most missingness on these items. Ethnicity was also significant, χ^2^(11, *N* = 3,906) = 83.51, *p* < .001, with “prefer not to say,” “other,” and “Asian/Asian British–Bangladeshi” showing the most missingness. In all chi‐square analyses, the majority characteristic had the lowest missingness except for sexual orientation, for which “gay/lesbian” showed the lowest.

## RESULTS

In the present sample, 49.3% of participants reported bullying or harassment, 66.5% reported verbal abuse, 16.3% reported experiencing physical assault, and 4.6% reported experiencing sexual assault. Regarding exposure to fatalities, 80.1% of the sample reported hearing about a fatality, whereas 30.3% reported seeing the aftermath of a fatality, and 12.0% reported witnessing a fatality.

Among all participants, 2.8% met the criteria for PTSD and 7.2% for CPTSD, for a combined total of 10.0%. Of individuals who were administered the ITQ, 6.8% met the criteria for PTSD and 17.5% for CPTSD, for a combined total of 24.3%. For the PHQ‐9, 27.9%, 29.4%, and 9.2% of participants scored in the mild, moderate, and severe symptom ranges, respectively. For the GAD‐7, 28.9%, 15.0%, and 14.2% scored in the mild, moderate, and severe symptom ranges, respectively.

### PTSD

Detailed model fit statistics for PTSD are available in Table 1. Steps 1–4 of the model imparted significant contributions to model fit. COVID‐19 variables in Step 5 and the total number of psychosocial hazard exposures in Step 6 did not significantly contribute to the model. The final model was significant, predicting 15.4% of PTSD cases and 98.8% of noncases. After controlling for all variables, exposure to bullying and harassment, hearing about a fatality, and witnessing a fatality were associated with PTSD. Covariates significantly associated with PTSD in the final model included the presence of a disability, higher scores on items related to work intensity, being employed at the company for less than 1 year, a higher total number of physical health problems, and a higher total number of sickness absences. Length of employment greater than 1 year and higher scores on the EWCS Working Life Perspectives subscale were associated with the absence of PTSD. All significant variable statistics are displayed in Table 2.

**TABLE 1:** Posttraumatic stress disorder (PTSD) and complex PTSD (CPTSD) model summaries

	PTSD				CPTSD			
Variable added	χ^2^	*df*	*p*	Nagelkerke's *R* ^2^	χ^2^	*df*	*p*	Nagelkerke's *R* ^2^
Predictors				.11				.10
Step	169.42	7	<.001		130.00	7	<.001	
Model	169.42	7	<.001		130.00	7	<.001	
Background/ demographic characteristics				.17				.19
Step	104.77	52	<.001		118.24	52	<.001	
Model	274.19	59	<.001		248.24	59	<.001	
Working conditions				.25				.28
Step	125.34	18	<.001		124.21	18	<.001	
Model	399.53	77	<.001		372.45	77	<.001	
Physical health				.27				.29
Step	29.03	6	<.001		20.83	6	<.001	
Model	428.56	83	<.001		393.27	83	<.001	
COVID‐19				.28				.31
Step	17.91	15	.268		18.51	15	.237	
Model	446.46	98	<.001		411.79	98	<.001	
Total exposures				.28				.31
Step	6.76	7	.455		8.65	7	.279	
Model	453.22	105	<.001		420.44	105	<.001	

*Note*: *N* = 3,197.

**TABLE 2:** Posttraumatic stress disorder logistic regression^a^

Variable	Wald χ^2^	*df*	*OR*	*p*	95% CI
Bullying/harassment	12.98	1	1.83	<.001	[1.32, 2.54]
Hearing about a fatality	5.77	1	1.78	.016	[1.11, 2.84]
Witnessing a fatality	10.57	1	2.10	.001	[1.34, 3.29]
Sector: Freight and nonpassenger services	4.43	1	2.27	.035	[1.06, 4.87]
Region: Southwest England	5.07	1	2.35	.024	[1.12, 4.95]
Disability (variable)	11.57	2		.003	
Disability present	11.10	1	1.88	.001	[1.30, 2.72]
Length of employment (variable)	11.57	4		.021	
1–5 years	9.74	1	0.30	.002	[0.14, 0.64]
6–10 years	6.56	1	0.37	.010	[0.17, 0.79]
11–20 years	9.86	1	0.29	.002	[0.13, 0.63]
≥20 years	9.55	1	0.27	.002	[0.12, 0.62]
Work intensity	31.68	1	1.79	<.001	[1.46, 2.19]
Working life perspectives	6.79	1	0.78	.009	[0.65, 0.94]
Total number of physical health problems	4.10	1	1.07	.043	[1.00, 1.13]
Past‐year number of sickness absences	23.97	1	1.01	<.001	[1.00, 1.01]
Mental well‐being change during COVID–19 pandemic (variable)	11.12	4		.025	

*Note*: *N* = 3,197.

^a^ Final model significant odds ratios (*OR*s).

### CPTSD

Detailed model fit statistics for CPTSD are available in Table 1. Steps 1–4 of the model significantly contributed to model fit. COVID‐19 variables in Step 5 the total number of psychosocial hazard exposures in Step 6 did not significantly contribute. The final model was significant, predicting 15.1% of CPTSD cases and 99.2% of noncases. After controlling for all variables, exposure to bullying or harassment, hearing about a fatality, and witnessing a fatality were associated with CPTSD. Covariates significantly associated with CPTSD in the final model included the presence of disability, higher scores on work intensity, being on a 0‐hr contract, and a higher total number of sickness absences. Higher scores on working life perspectives were associated with the absence of CPTSD. All significant variable statistics are displayed in Table 3.

**TABLE 3:** Complex posttraumatic stress disorder logistic regression^a^

Variable	Wald χ^2^	*df*	*OR*	*p*	95% CI
Bullying/harassment	11.52	1	2.02	.001	[1.35, 3.03]
Hearing about a fatality	3.97	1	1.77	.046	[1.01, 3.09]
Witnessing a fatality	4.73	1	1.79	.030	[1.06, 3.02]
Sector: Freight and other nonpassenger	5.74	1	3.10	.017	[1.23, 7.84]
Staff group: Train support services	5.90	1	3.11	.015	[1.25, 7.75]
Job security: Insecure/0‐hr contract	4.72	1	2.56	.030	[1.10, 5.98]
Region: Southwest England	4.34	1	2.61	.037	[1.06, 6.45]
Disability (variable)	13.73	2		.001	
Disability present	13.46	1	2.20	<.001	[1.44, 3.35]
Length of employment (variable)	10.57	4		.032	
1–5 years	8.50	1	0.27	.004	[0.11, 0.65]
6–10 years	5.82	1	0.33	.016	[0.13, 0.81]
11–20 years	9.54	1	0.24	.002	[0.09, 0.59]
≥20 years	7.57	1	0.25	.006	[0.09, 0.67]
Work intensity	24.26	1	1.83	<.001	[1.44, 2.33]
Working life perspectives	10.73	1	0.69	.001	[0.56, 0.86]
Past‐year number of sickness absences	15.98	1	1.01	<.001	[1.00, 1.01]
Mental well‐being change during COVID–19 pandemic (variable)	13.69	4		.008	

*Note*: *N* = 3,197.

^a^ Final model significant odds ratios (*OR*s).

### Depressive symptoms

Detailed model fit statistics for depression are available in Table 4. Steps 1–5 of the model contributed significantly to model fit. The total number of psychosocial hazard exposures in Step 6 did not significantly contribute to the model. The final model explained 46.4% of the variance. After controlling for all variables, none of the psychosocial hazards were associated with PHQ‐9 scores. In the final model, female sex, being single or divorced, the presence of a disability, workplace physical ergonomics, work intensity, employment length of less than 1 year, the total number of physical health problems, and a physical health self‐rating of average or below were positively associated with PHQ‐9 score. Age and scores on the EWCS Social Environment and Working Life Perspectives subscales were negatively associated with PHQ‐9 scores. All significant associations are displayed in Table 5.

**TABLE 4:** Patient Health Questionnaire–9 (PHQ‐9) and Generalized Anxiety Disorder–7 (GAD‐7) linear regression model summaries

	PHQ‐9					GAD‐7				
Variable added	Adjusted *R* ^2^	Δ*F*	*p*	Δ*f* ^2^	Total *f* ^2^	Adjusted *R* ^2^	Δ*F*	*p*	Δ*f* ^2^	Total *f* ^2^
Predictors	.08	41.94	<.001	.09	.9	.07	36.56	<.001	.08	.08
Background/ demographic characteristics	.17	7.66	<.001	.12	.21	.16	7.10	<.001	.11	.19
Working conditions	.36	51.60	<.001	.23	.56	.33	46.93	<.001	.21	.50
Physical health	.42	54.34	<.001	.06	.72	.36	22.14	<.001	.03	.56
COVID‐19	.46	18.69	<.001	.05	.87	.41	19.75	<.001	.06	.70
Total number of exposures	.46	1.13	.340	<.01	.87	.41	1.22	.286	<.01	.70

**TABLE 5:** Patient Health Questionnaire–9 linear regression^a^

Variable	*B*	β	*t*(3,091)	*p*	95% CI	*f^2^*
Staff group						
Operations and customer services	1.40	.07	3.65	<.001	[0.65, 2.15]	<.01
Train support services	1.63	.05	2.91	.004	[0.53, 2.73]	<.01
Infrastructure	1.82	.11	4.41	<.001	[1.01, 2.63]	<.01
Other staff groups	1.65	.08	3.81	<.001	[0.80, 2.49]	<.01
Sex: female	0.50	.03	2.12	.034	[0.04, 0.96]	<.01
Marital status	0.86	.05	3.27	.001	[0.35, 1.38]	<.01
Single						
Divorced or separated	1.57	.05	3.84	<.001	[0.77, 2.37]	<.01
Disability present	0.75	.03	2.42	.016	[0.14, 1.35]	<.01
Age (years)	−0.06	−.10	−5.97	<.001	[−0.08, −0.04]	<.01
Physical ergonomics of work environment	0.07	.05	2.74	.006	[0.02, 0.11]	<.01
Work intensity	0.10	.20	9.71	<.001	[0.08, 0.12]	.02
Social environment at work	−0.15	−.08	−3.93	<.001	[−0.22, −0.08]	<.01
Working life perspectives	−0.43	−.17	−8.64	<.001	[−0.53, −0.33]	.01
Employment length <1 year	1.36	.04	2.48	.013	[0.29, 2.44]	<.01
Total number of physical health problems	0.37	.14	8.74	<.001	[0.29, 0.45]	.01
Past‐year number of sickness absences	0.01	.06	4.20	<.001	[0.01, 0.01]	<.01
Physical health						
Very poor	3.48	.09	5.98	<.001	[2.34, 4.62]	<.01
Poor	2.65	.14	8.85	<.001	[2.07, 3.24]	.01
Average	0.91	.07	4.29	<.001	[0.50, 1.33]	<.01
Mental well‐being during COVID–19 pandemic						
Somewhat better than before	−0.79	−.03	−2.09	.036	[−1.52, −0.05]	<.01
Somewhat worse than before	1.16	.08	5.57	<.001	[0.75, 1.56]	<.01
Significantly worse than before	4.36	.23	15.03	<.001	[3.79, 4.93]	.04

*Note*: ^a^Final model significant beta coefficients.

### Anxiety symptoms

Detailed model fit statistics are available in Table 4. Steps 1–5 of the model conferred significant contributions to model fit. The total number of psychosocial hazard exposures in Step 6 did not significantly contribute to the model. The final model explained 41.2% of the variance. After controlling for all variables, exposure to bullying or harassment was positively associated with GAD‐7 scores, whereas hearing about a fatality was negatively associated. In the final model, identifying as bisexual, being divorced, having a disability, being on a 0‐hr contract, higher scores on the EWCS Physical Environment, Physical Ergonomics, and Work Intensity subscales, a higher total number of physical health problems, and a physical health self‐rating of average or poor were positively associated with GAD‐7 scores. All significant associations are displayed in Table 6.

**TABLE 6:** Generalized Anxiety Disorder–7 linear regression^a^

Variable	*B*	β	*t*(3,091)	*p*	95% CI	*f^2^*
Bullying/harassment	0.40	.04	2.16	.031	[0.04, 0.77]	<.01
Hearing about a fatality	−0.47	−.03	−2.08	.038	[−0.92, −0.03]	<.01
Staff group						
Operations and customer services	1.53	.08	4.35	<.001	[0.84, 2.21]	<.01
Train support services	1.52	.05	2.97	.003	[0.52, 2.53]	<.01
Infrastructure	1.60	.11	4.22	<.001	[0.86, 2.34]	<.01
Other staff groups	1.56	.09	3.96	<.001	[0.79, 2.34]	<.01
Job security: Insecure/0‐hr contract	1.32	.03	2.14	.032	[0.11, 2.54]	<.01
Sexuality: Bisexual	0.97	.03	2.12	.034	[0.07, 1.87]	<.01
Marital status: Divorced/separated	0.78	.03	2.09	.037	[0.05, 1.52]	<.01
Disability present	0.63	.03	2.23	.026	[0.08, 1.18]	<.01
Age (years)	−0.07	−.13	−6.90	<.001	[−0.08, −0.05]	<.01
Physical environment at work	0.03	.05	2.42	.016	[0.01, 0.06]	<.01
Physical ergonomics at work	0.05	.04	2.37	.018	[0.01, 0.09]	<.01
Work intensity	0.11	.25	11.68	<.001	[0.09, 0.13]	.03
Social environment at work	−0.16	−.10	−4.57	<.001	[−0.23, −0.09]	<.01
Working life perspectives	−0.29	−.13	−6.40	<.001	[−0.38, −0.20]	<.01
Employment length						
<1 year	1.55	.05	3.09	.002	[0.57, 2.54]	<.01
6–10 years	0.69	.04	2.39	.017	[0.12, 1.25]	<.01
Total number of physical health problems	0.25	.11	6.53	<.001	[0.18, 0.33]	<.01
Past‐year number of sickness absences	0.01	.08	5.31	<.001	[0.01, 0.02]	<.01
Physical health self‐rating						
Poor	0.76	.05	2.77	.006	[0.22, 1.30]	<.01
Average	0.44	.04	2.25	.025	[0.06, 0.82]	<.01
Mental well‐being during the COVID–19 pandemic						
Somewhat worse than before	1.22	.10	6.42	<.001	[0.85, 1.59]	<.01
Significantly worse than before	4.32	.26	16.29	<.001	[3.80, 4.84]	.05

*Note*: ^a^Final model significant beta coefficients.

## DISCUSSION

The current study provides the most comprehensive assessment of mental health in the U.K. rail workforce to date. The rate of depression and anxiety was high in this sample compared with a large general population cohort study during the COVID‐19 pandemic, which found that 31.6% and 26.0% of participants scored in the moderate to severe categories for PHQ‐9 and GAD‐7, respectively (Jia et al., 2020). The rate of PTSD in the present sample among participants who were administered the ITQ was also high in comparison to an international COVID‐era meta‐analysis, which found a 15% prevalence for all forms of PTSD (Zhang et al., 2021). The frequency of exposure to different psychosocial hazards was also striking. The present findings demonstrate that there is a high burden of mental distress, as well as a high rate of exposure to significant occupational stressors, in the rail workforce.

Our first hypothesis, that psychosocial hazards would be associated with scores on the mental health outcome measures of interest, was partially supported. None of the main exposures were associated with PHQ‐9 scores in the final model, although exposure to bullying and harassment was positively associated with GAD‐7 scores; however, the effect was small. The effect sizes for bullying and harassment, hearing about a fatality, and witnessing a fatality were much higher with respect to PTSD and CPTSD. This pattern of difference could reflect that PTSD is typically precipitated by a discreet event or series of events, whereas individual etiologies of depression may involve a range of smaller biopsychosocial influences. However, the findings conflict with substantial evidence linking workplace bullying to depression (e.g., Verkuil et al., 2021) as well as evidence linking trauma exposure to comorbidities of PTSD and both depression and anxiety (e.g., Johansen et al., 2013). Despite this and the small effect demonstrated in the GAD‐7 model, the finding that exposure to bullying and harassment was independently significant in three of the four models is interesting and may be of relevance to clinical practice and any future mental health initiatives in the U.K. rail industry.

The finding that hearing about a fatality was negatively associated with GAD‐7 scores was unexpected, although the effect was small. Possible explanations could include confounding; it is possible that working in supportive and communicative environments makes hearing about a fatality more likely. There is evidence that perceived support within an organization serves as a protective factor following critical incidents (Gouweloos‐Trines et al., 2017). That said, the effect of hearing about a fatality was different for the other outcomes, as this exposure was associated with a higher odds ratio for PTSD.

The second hypothesis, that the total number of psychosocial hazards exposures would be independently associated with mental health outcomes, was not supported. This finding is unexpected given evidence reported elsewhere (e.g., Martin et al., 2013). However, it should be noted that the data on these items were potentially unreliable due to the use of retrospective reporting.

The third hypothesis was that working conditions would contribute to the association between hazard exposure and outcomes. Working conditions made significant contributions to all four models. For depression and anxiety, physical ergonomics and work intensity appeared to be important variables. Although both variables demonstrated small effect sizes, the effect for work intensity was stronger than for bullying and harassment. Social environment, again with a small effect size, appeared to be a possible protective factor, which echoes previous studies (Park et al., 2004; Plaisier et al., 2007). Evidence from a systematic review suggests that high demand and low decision latitude are the strongest workplace environment predictors of depression (Bonde, 2008), which could explain why we observed a stronger effect for work intensity as compared to bullying and harassment.

Scores on the EWCS Social Environment subscale were not associated with PTSD or CPTSD, which is inconsistent with substantial evidence regarding social support, risk, and resilience (e.g., Brooks et al., 2019; Lauth‐Lebens & Lauth, 2016; Somville et al., 2016). This could partly be explained by the correlational nature of the data. An employee who reports that their workplace is generally supportive may not necessarily have benefitted from good‐quality social support following a specific traumatic event. However, higher scores on the EWCS Working Life Perspective subscale were associated with an absence of both outcomes. This subscale characterizes aspects of job satisfaction, which has been found elsewhere to be protective against PTSD (Somville et al., 2016). In addition to weaker positive associations with PHQ‐9 and GAD‐7 scores, work intensity was associated with moderately higher odds ratios for PTSD and CPTSD in all final models. Similar to bullying and harassment, work intensity was an important variable in this sample. These results support other examples in the literature concerning job burnout (e.g., De Lange et al., 2004; Dengping, 2020), which have demonstrated that work intensity is associated with burnout and, subsequently, poorer mental health.

There were some limitations to the study that should be discussed. This was a cross‐sectional, remotely delivered survey. The study did not include any longitudinal data and was correlational; as such, causality cannot be implied. Nearly one in five participants failed to complete all measures and were excluded from the analyses. The data were unlikely to be missing completely at random, and there were several significant chi‐square tests with regard to missing data for demographic variables. Therefore, it is likely that there is a subset of participants, perhaps more likely those with minority characteristics, to whom our analysis cannot be generalized. In addition, the response rate was relatively low when considering the size of the population. Finally, the COVID‐19 pandemic has likely had a substantial impact on mental health (Cullen et al., 2020; Pereira‐Sanchez et al., 2020) along with considerable changes to the effects of workplace stress on employees (Kniffin et al., 2021). Reported changes in mental well‐being during the pandemic were significantly associated in all models, which likely contributed to some of the observed variance as well as to the relatively high rates of PTSD, CPTSD, depression, and anxiety in the sample.

As the largest study of its kind in this population to date, the present work documents extensive exposure to both psychosocial hazards and traumatic events among rail industry employees and highlights the widespread mental health outcomes, including PTSD and CPTSD, in this population, with some evidence of associations between these exposures and outcomes. The findings suggest a potentially high burden of mental distress in this population along with a number of important and potentially modifiable risk factors for PTSD, CPTSD, depression, and anxiety, which can guide the management of mental health in the workplace. Given the potentially high rates of mental distress, it appears relevant to suggest future investigations of stepped‐care initiatives in this area, perhaps based on models such as trauma risk management (Whybrow et al., 2015). This would, ideally, involve population‐level interventions, such as mental health awareness training; targeted interventions, such as support following potentially traumatic incidents; and screening employees most at risk, with processes in place to support them, with consent, in seeking treatment through local services. Other potential avenues could include initiatives to place mental health professionals within rail organizations to provide support to front‐line colleagues, provide liaison or advocacy services, or both.

The present findings suggest that working conditions, especially the emotional impact of work and the stress involved with dealing with the public, are risk factors that can be taken into account when designing mental health initiatives for railway companies. Along with these, a number of covariates that were not specifically named in our hypotheses also appear to be important potential risk factors, such as the presence of a disability, a finding that is echoed elsewhere (Scott et al., 2009; Turner et al., 2006), and the total number of physical health problems, which has also appeared in previous studies (e.g., Meader et al., 2011). These and other factors are worthy of future investigation with the present data or future samples.

## OPEN PRACTICES STATEMENT

The study reported in this article was not formally preregistered. Neither the data nor the materials have been made available on a permanent third party archive; requests for the data or materials can be sent via email to the lead author at Laurence_carnall@hotmail.co.uk.

## AUTHOR NOTE

During this project, Laurence Carnall was a trainee psychologist for the University of Surrey; he no longer studies or works under this institution.
